# Plasmonic Sensors Based on Doubly-Deposited Tapered Optical Fibers

**DOI:** 10.3390/s140304791

**Published:** 2014-03-10

**Authors:** Agustín González-Cano, María-Cruz Navarrete, Óscar Esteban, Natalia Díaz-Herrera

**Affiliations:** 1 Applied Optics Complutense Group, Facultad de Óptica y Optometría, Universidad Complutense, Arcos de Jalón 118, Madrid 28037, Spain; E-Mail: ndiazher@fis.ucm.es; 2 Applied Optics Complutense Group, *Facultad* de Ciencias Físicas, Universidad Complutense, Ciudad Universitaria, Madrid 28040, Spain; E-Mail: mcnavarr@fis.ucm.es; 3 Grupo de Ingeniería Fotónica, Universidad de Alcalá de Henares, Alcalá de Henares 28871, Spain; E-Mail: oscar@depeca.uah.es

**Keywords:** surface plasmon resonance, tapered optical fibers, refractometry

## Abstract

A review of the surface plasmon resonance (SPR) transducers based on tapered fibers that have been developed in the last years is presented. The devices have proved their good performance (specifically, in terms of sensitivity) and their versatility and they are a very good option to be considered as basis for any kind of chemical and biological sensor. The technology has now reached its maturity and here we summarize some of the characteristics of the devices produced.

## 1. Applied Plasmonics and the Devices that Make It Possible

### 1.1. Some General Considerations

It is almost impossible to be comprehensive or exhaustive when speaking of the many devices based on the excitation of plasma waves that have been presented in the last decades in the literature. Even to try to classify the many applications and variations of these devices is a very complex task.

We can rely, then, on some major features and concepts to help us introduce some order in the discussion. *Plasmonics* necessarily imply that we must be able to produce a so-called plasma wave under appropriate conditions, which leads us to the use of *metals*. To generate plasmons in these metals we cannot use conventional, common arrangements, since the conditions of *coupling* with the plasma waves are restrictive. In the successively published papers, varied mechanisms have been proposed to provide a good coupling between an incident, conventional (guided or not) electrical wavefield and the surface plasma waves to be generated in the selected material.

One major goal, then, of *Applied Plasmonics* is to achieve the highest *efficiency* in this plasmon excitation process. While other approaches to plasma waves are more centered in the basic phenomenology of wave propagation in material media or the theoretical formulation of that propagation, we focus in the effective generation of plasmons in the desired ranges and conditions of measurement, and this is the key feature for any applied plasmonic devices.

Nowadays, one very important field of application of plasmonic devices is chemical and biological sensing. The researchers working in this field tend to prefer surface plasmon resonance (SPR) devices based on evanescent coupling with thin layers obtained via the phenomenon of *attenuated total reflection* (ATR), and, in the scientific publications devoted to chemo- and biosensors the setup used to provide the plasma waves is only rarely discussed, assuming that it is always of this kind. It is true that today we can use commercial systems based on ATR with very good performance and that chemists and biologists can then concentrate in the research on what to *add* to this basic setup, namely, the recognition agents and the chemical or biological processes involved in the detection of the desired analyte.

However, ATR is in no way the only way to provide evanescent coupling, and there exists a quite active field of research that focuses its interest on the development of new concepts and setups to excite plasmons, with an improved performance of the transducers to face the always increasing exigencies of chemical analysis and biotechnology. Other principles of measurements or other configurations have been tried in recent years, and very good results have been reported in the literature, but it seems that somehow the transference of the basic knowledge produced by the groups working in this direction to the community of users of SPR technology in the biosensing field is not as good as it should, which explains that sometimes very interesting proposals of new conceptions of plasmon generating devices with astonishingly high quality indicators have not been taken into account, if not to replace the existing technology, at least to complement it. Here we discuss some of these different possibilities, which are based on the use of waveguides of a particular kind.

### 1.2. SPR Sensors Based on Waveguides

We can achieve evanescent coupling with guiding devices in varied ways. If we limit our discussion to *optical fibers*, many devices have been reported based on evanescent coupling between the light guided by the fiber and one or more layers deposited on the guide. Optical fibers are ubiquitous, cheap, show great performance, their technology is completely mature and the assisting elements such as couplers, connectors, *etc.* are easily accessible. Optical fibers are first a channel that carries the interrogating beam to the sensing area, but they can also be modified to provide a coupling mechanism with plasmons.

We can then think, thanks to the use of optical fibers, of a full integration of all the elements required in the sensing process, from the light source to the detector in a, so to speak, closed circuit, where the efficiency in the light propagation and collection is maximized. We can also maintain this efficiency over many meters or even kilometers and therefore access distant locations (sometimes only reached with difficulty or even danger) without the need of displacing most of the elements of the measuring setup and maintaining a stable installation that processes continuously in real time, perhaps in a distributed or quasi-distributed way. Many different sensors based on fibers have been depicted, and optical fiber technology is the preferred option in quite demanding fields, such as structural monitoring. In what respects to SPR sensing, the use of optical fibers is already old and many sensors have been proposed, with very good results. We could say that all the measurements made today with ATR-based devices could be made in principle with fiber optic sensors (FOS), in many cases with the added advantages associated to fibers. However, as we have said before, it seems that these good characteristics are not always sufficiently taken into account by potential users.

To develop a real SPR-FOS we must perform an in principle contradictory operation, namely, to make a guided wave interact with something outside the waveguide, that is, to somehow subvert the main reason why fibers are useful most of the time: their capacity of transporting light at long distances without losses. Most commonly this is made by modifying the fiber to permit the access to the evanescent field of the guided modes. This field is put then in contact with the metal layer that must be excited.

One obvious way of doing it is by reducing the thickness of the fiber cladding. Of course this is a process that involves a compromise, since we still want the wave guided after the interaction with the studied medium, so we can conduct it to the detector via the same guide. But in some cases this can be done while at the same time ensuring good access to the evanescent field. The direction of the wave vector of this field is the right one to provide coupling with surface plasma waves, so we can deposit the metallic layer on the reduced cladding and, given some coupling conditions, produce SPR. The reduction of the thickness of the cladding is made by chemical etching or polishing, most commonly. Layers then are flat and are usually deposited in a vacuum chamber controlling the thickness of the deposited metal (gold is the most used one). One can find many results in the literature of sensors based on that principle, and in the past our group showed the feasibility of salinity measurements with a SPR sensor made with a polished fiber and a double deposit (more on the double deposit approach in the next section), even under real measuring conditions, with *in situ* experimental measurements performed in a research vessel [1,2].

### 1.3. Use of Tapered Fibers

We have said that coupling efficiency is one of our main goals, and, for this, there is a better way to provide access to the evanescent field of a fiber: tapering it. It is better because the symmetry of the guide is maintained, and also because we can easily increase the size of the interaction region, and have more degrees of freedom in the design of the devices.

We will describe the tapering process and the resulting tapered fibers in the next section, since this review is centered in the SPR sensors based on that kind of elements, but it is important to note here that, first, this tapering process is very easy and does not require any sophisticated apparatus (it is also very repeatable and easily automatized) and, second, that, when using tapered fibers as substrate for the subsequent deposition of the layers where plasmons are to be generated, those layers are not going to be flat, which is, as we will see, very interesting in terms of performance, although it also supposes a certain challenge for their fabrication.

### 1.4. Some Words on the State-of-the-Art

The main objective of this paper is to provide a general view of the advantages achieved when tapered fibers are used as basis of SPR sensors and not to try to cover all the above mentioned fields of SPR sensing. Some very comprehensive and excellent reviews have already been published that can provide the required information [3–5]. However, it is convenient that we somehow establish some means of comparison between these different options to justify the usefulness of tapered-fiber-based SPR sensing.

The discussion tends to be centered (abusively, in some cases) on the *sensitivity* of the sensors, taking for that the slope of the curves that relate the changes of the refractive index with the displacement of the plasmon dips. Here, it is common to find in the literature some works that perform only theoretical simulations and establish unrealistic figures. Also, in some other cases the values of sensitivity have been calculated starting from measurements in perfect conditions in the lab and do not take into account, frequently, other effects that can affect the measurements in a great way, like temperature. In that sense, sensitivities as high as 10^−7^ RIU have been reported, but in principle, 10^−5^ is a more realistic estimation, which is still incredibly good, and probably as high as one can get in refractometry. Our sensors, as it will be seen below, can easily reach experimental values of sensitivity in the range of thousands of RIU/nm, which implies that, even with a modest resolution of the spectrometer used in the measurements, we are comfortably placed in the range of maximum operative sensitivities. This is even increased when using InN as dielectric deposited material. Again, these are laboratory values and they can also be subject to discussion, but the main message is that shifting from the traditional options to tapered fibers does not decrease the performance of the device.

When used as chemo- or biosensors, the limits of detection and the resolution are important parameters, and many papers discuss them, but, in that case, the response of the system depends on the mechanism employed to make selective the response, the recognition agents used. What we propose here is the immediate step before this adding of the agent and the, so to speak, basal sensitivity as a refractometer of tapered-fiber-based SPR sensors is perfect to ensure a very good performance in terms of analyte detection.

Other aspects have been mentioned before, but they are not always taken into account by the authors when presenting their results. If we need to work *in situ*, in real time, with prolonged measuring times, in hazardous places, *etc.*, any setup based in elements such as prisms, that must incorporate mechanisms for angular interrogation and polarization-controlling elements are not competitive, and can even be unusable. The good possibilities that fibers offer apply here, and the small size and extreme simplicity of our arrangements permits to think in highly integrated, versatile and robust devices, that can allow multiparametric, accurate and fast measurements and can adapt themselves to the concept of lab-on-a-chip.

To summarize, the state-of-the-art of SPR transducers based on the already existing technologies is that of a very mature technology, completely accepted by the analytical community which centers its interest in the adaptation of these setup to their needs of detection of a particular analyte. But, at the same time, there are other options, which are clearly more advantageous in several aspects that should be more exploited for the same goal of development of chemo- and biosensors, especially for some exigent measurement conditions, within which the use of tapered optical fibers have proved its validity. We will give now some indications, in the next sections of the variety of devices that have been produced and the ranges that they can cover, attending to the publications from by our group in, roughly, the last fifteen years, trying to be concise and systematic.

## 2. Tapering Fibers: Some Considerations on the Fabrication Process

The most usual setup to produce tapered fibers is the so-called *travelling burner technique*: a section of the fiber is heated by a burner displaced in an oscillating way in a trajectory longitudinal to the fiber and at the same time, and in this same direction, the fiber is gently pulled by two motors. The amplitude and frequency of the movement of the burner determine the characteristics of the tapered region, namely, its length, the diameter of the waist of the tapered area and also the profile of the transition region between this waist and the unaltered fiber portions before and after the taper. These parameters can easily be controlled and the fabrication process is highly repeatable.

The tapers produced with this technique are called *uniform-waist tapers* (UWTs), in comparison to other type of also very common tapers called *biconical* (BTs), which are usually fabricated with a fiber splicer. UWTs are considerable more adiabatic than BTs, because the light in them is smoothly coupled between the different regions of the taper, while in BTs the transition is much more abrupt and the losses are quite high. Also, the travelling burner setup permits a better control of the fabrication parameters than with BTs [6].

**Figure 1.**
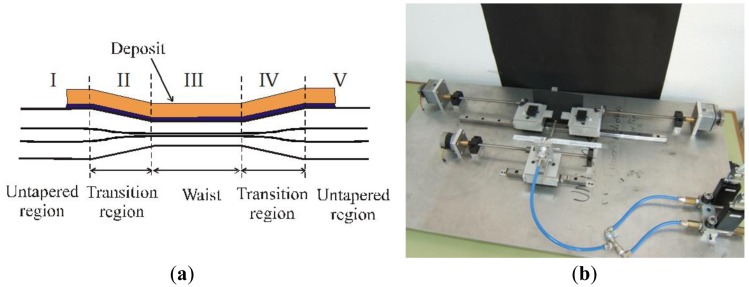
(**a**) A scheme of a uniform-waist taper, and its different regions; (**b**) A photograph of the fabrication setup.

One characteristic of UWTs extremely relevant in terms of their use as SPR devices is that cylindrical symmetry is always preserved in them. If we polish or etch a fiber to reduce its cladding, we drastically transform its geometry, which has huge impact in its guiding properties, but with UWTs we can achieve an almost perfect coupling between the three cylindrical guides involved: the two unaltered areas (regions I and V of Figure 1a), where the core “contains” the field with a little expansion of the evanescent field in the cladding area (we use almost always single mode fibers, so we can visualize guided light by thinking in that mode guided by the core) and region III, where the tapering process has resulted in the effective disappearance of the core and the creation of a cladding-air multimode guide, and where the evanescent field is easily reachable. In between these guides we have the transition regions that, when they have the right profile, provide adequate coupling with very low losses. Of course, the guiding characteristics of the waist depend on its diameter, but, as one can easily imagine, the smaller this diameter is, the more evident are the effects associated to the tapering, in particular the one we are interested in: evanescent coupling.

We have tested many different combinations of waist length, total taper length and waist diameter. It is not really critical for the development of SPR sensors to force in an extreme way the tapering process. Waists of the order of 30 μm (starting from conventional single-mode fibers in the wavelength region around 800 nm) are more than enough to provide spectacular evanescent coupling and subsequently very strong resonances. The process is fully automatized and highly controllable and repeatable, as it is proven by the hundreds of tapers produced, and the agreement between the predictions concerning the final geometry of the taper starting from the fabrication parameters and the actual form of the produced tapers is extremely good. Once one has the travelling-burner scheme functioning (some aspects, such as the control of the fluxes of the gases of the burner, are important here), production of tapers implies no problem at all, and many different kind of fibers can be used with the same machine. A photograph of the setup is shown in Figure 1b.

## 3. Generating a SPR Sensor: The Deposition of Materials

Once we have the taper we must deposit the layers required to generate the plasmon excitation in the tapered region. In principle it is not exactly equivalent to depositing them only in the waist (region III) or on the whole taper (regions II + III + IV), but, again, the final result in terms of performance is not critical. However, it is obvious that it is not the same thing in any way to deposit layers on a flat surface, as it happens with polished fibers, that here, where the substrate is still cylindrical. Being in principle a disadvantage, it is on the contrary the fact that makes SPR sensors based on UWTs really special. This is so because of the following features:

- Layers are no longer flat, and a thickness gradient is imposed here. Thickness of the layers is a most critical parameter for SPR, so what we have in principle is the possibility of different ways of fulfilling the excitation conditions. This will produce multiple resonances [7].
- For the same reason, the strong dependence of plasmon excitation on the polarization of the incident field is diminished, because we can no longer strictly define directions of vibration with respect to the plane of the layers (there is no such “plane” any more). In fact, this dependence can even be completely eliminated if we produce a symmetrical deposit. This is a most remarkable fact, since, as we have said, if one has a really representative feature of plasmons is this dependence on polarization (only TM fields can excite plasmons) [8].

We have experimentally demonstrated these two unusual behaviors of plasmons, thanks to the use of UWTs. They are not to be found in any of the other existing options for SPR, especially in those based on ATR. In our opinion, this is a very important fact not only from the point of view of the applications, but also from the most basic point of view of the understanding and clarifying the dynamics of plasma wave excitation. This enrichment of phenomenology has very important practical consequences.

Before we can present experimental evidence of these facts we must say something about the materials employed for the deposited layers, their choice and the range of thicknesses we must use (and the way we select them). The very use of the plural form when speaking of *layers* is interesting in itself. Most commonly, a single layer of gold (more rarely, of other metals such as silver or aluminum) is employed in SPR devices. The excitation of plasmons in that layer occurs if some conditions are fulfilled, that link the refractive index of the substrate, the layer and the outer medium, and the thickness of the layer. All of this is well-known and it is not necessary to give more details: it is what we find, for instance, in commercial devices.

However, we have proved that a different combination of deposited materials permit to tune in a much wider dynamic range the behavior of the system, as it is shown in Section 5. We have been using most commonly a combination of a metal (aluminum, some nanometers to some tenths of nanometers thick) and a dielectric (titanium dioxide, and more recently, indium nitride, tenths of nanometers thick), calculating in each case which thicknesses must be deposited to permit the detection for different spectral ranges and values of the refractive index of the surrounding medium. These doubly-deposited devices (DLUWTs, *double-layer uniform-waist tapers*) are nowadays a consolidated technology for providing SPR sensing and have proven their very good behavior and performance. The rest of the paper will be devoted to summarize the main results obtained with DLUWTs.

## 4. Experimental Results I: Influence of the Geometry of Deposition and the Dependence on Polarization

We are only going to present representative results and therefore we will place the emphasis on the specific added value of DLUWTs with respect to the other existing devices. For that reason we show first how, as said above, the particularity of the non-flat geometry of the deposited layers influences the performance of the transducers [7].

**Figure 2.**
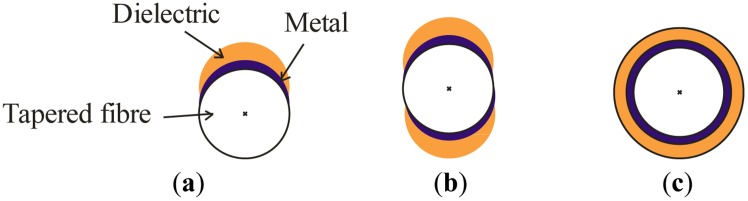
Different options for the geometry of the deposit on the tapered region. (**a**) asymmetric; (**b**) double-sided; (**c**) symmetric.

**Figure 3.**
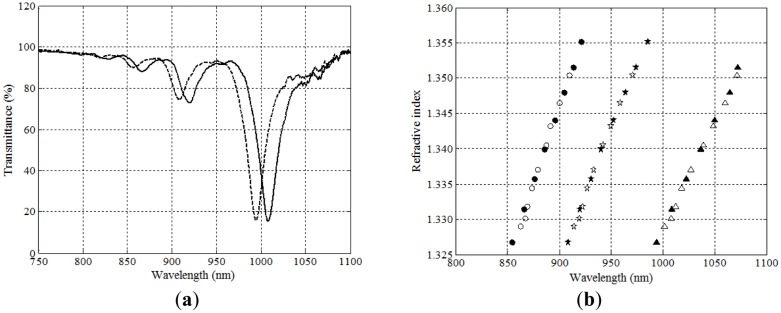
Representative experimental behavior of asymmetrically deposited DLUWTs. On the left (**a**), spectral transmittance, showing several plasmon dips, which displace themselves when changing outer refractive index; On the right (**b**) these displacement in terms of the value of plasmon wavelength.

In Figure 2 we show the three main possibilities for depositing layers on a taper, in a sectional view. In the first case, Figure 2a, we speak of “asymmetric” deposition. In principle, we can consider that, at least for some part of the deposited layers, we could take a representative, average value of the thickness, and assume that this is not too different to a flat-layer deposition. In fact, this is the way we can easily choose which thicknesses we desire to deposit, starting from very simple simulations that can predict the region of refractive indices for which the sensor will be operative. This gives, let us say, the “main plasmon” of the structure, and we have shown how it is easily tuned to the desired operating region. For instance, in Figure 3a (where we show the spectral transmittance of the device), we are working in the near infrared region and with typical thicknesses of 8–10 nm of aluminum and 40–50 nm of titanium dioxide. If we were working with really flat layers this should be the only plasmon excited, but, as it can easily be seen in the figure, this is not the case anymore with asymmetric depositions. We can observe very well defined dips that displace the same way plasmons do when the refractive index of the outer medium is changed. That means that we have *multiple sensor resonances*, a feature specific of asymmetric DLUWTs.

The possibility of counting with more than one plasmon for the measurements is very interesting from the practical point of view, both for self-referencing and for multiparametric detection. It is also an experimental confirmation of the fact that a gradient of thickness in the deposited layers provides not only one, but several fulfillments of the conditions of resonance, but, obviously, for different plasmon wavelengths. Following the displacement of those wavelengths we can measure refractive index with a remarkably high sensitivity (Figure 3b), although even higher ones can be achieved in some other cases, as we will see below.

However, another thing that we can observe with asymmetric (and not with flat) deposits is that the dependence on polarization of plasmon excitation decreases. The fact that this dependence is, so to speak, inherent to the conditions of excitation imposes the need of adding polarization controlling elements to any experimental setup, if one wants to make the response of the device stable and optimal. This is always inconvenient, because the final configuration of the sensor is complicated. We have been using this kind of polarization control (normally, Lefébvre loops) and in the case of asymmetrical deposits we showed how the use or non-use of these elements was almost irrelevant, proving that the excitation of plasmons was no longer critically dependent on that, because, as we have said, the deposit extends itself in a larger angle, and fields vibrating in a direction other than the normal can produce resonances.

**Figure 4.**
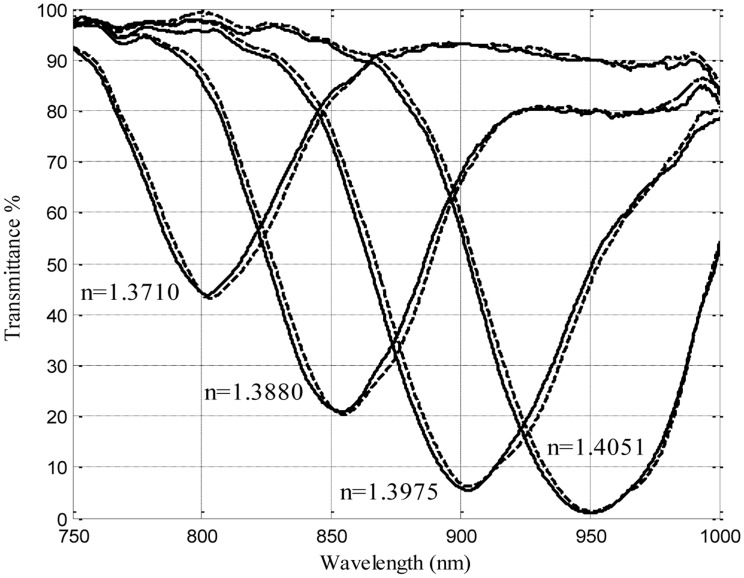
Representative experimental behavior of symmetrically deposited DLUWTs: a wide plasmon dip, that displaces itself when refractive index of the outer medium changes and no polarization dependence (continuous and dashed lines show use or not use of controlling elements (originally published in [8] and reproduced by kind permission of Springer Science + Business Media).

A natural way of extending this property was to produce fully symmetrical deposits, in which the cylindrical symmetry of the guide was still preserved once the layers are present. To achieve this we needed to introduce in the vacuum chamber a rotating mechanism to expose the whole circumference of the fiber, and the results obtained were remarkable, since no dependence with polarization was present at all (and hence there was no need of the addition of any controlling elements). Another remarkable feature of symmetrical DLUWTs is that, as expected, we had no longer multiple resonances, but only one, quite wide, dip (Figure 4). Since we have no preferred direction of vibration for the excitation and the thickness was similar in the whole section of the taper, all the resonances were, so to speak, the same. The width is associated to the residual gradient of thickness, unavoidable because the strict homogeneity of the deposit was unreachable even rotating the sample. Again, the results were important not only because of their potential usability (the fact that we will not need any more polarization controllers is a very interesting practical improvement) but also because of the insights that they provide to the way plasmons are generated in this kind of structures.

In that sense, the fact that the structures based on up and down deposits (type b of Figure 2) show an increased dependence on polarization corroborates the idea that this dependence is associated to privileged directions of vibration.

## 5. Experimental Results II: Possibility of Wavelength Tuning in a Wide Spectral Area

With DLUWTs, and only by changing the thicknesses of the deposited layers (not even changing the materials) we can displace the operating region of the devices in a range of about 1,000 nm. We have been working most of the time in the near infrared area (around 850 nm) and for values of the measured refractive index corresponding to those of aqueous media (around 1.33), the most important one for biological applications. We have proven that we can work, for the same range of refractive indices, in the C-Band with wavelengths around 1500 nm (no single-layer device can do that) [9], or with wavelengths well into the visible region, down to 500 nm [10], and always with the same good behavior in terms of simplicity and sensitivity. This versatility is achieved at almost no cost, since the production of the devices is strictly the same.

In recent times we have been studying a more ambitious step that will carry SPR technology well into the mid infrared region, although it seems that integrated waveguides are then the best option [11]. However, fibers can still be used for wavelengths longer than those of the communications band.

**Figure 5.**
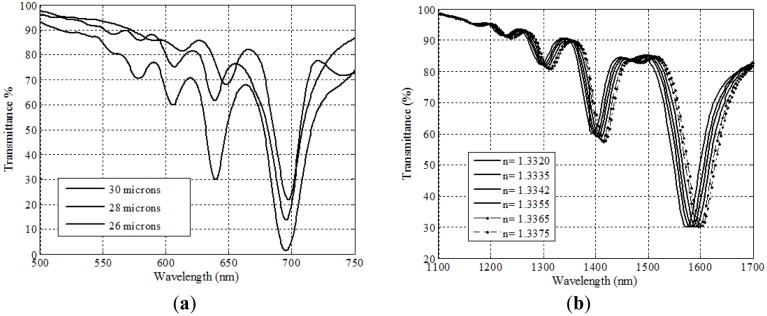
Representative experimental behavior DLUWTs working in extreme spectral regions for aqueous outer media. (**a**) Visible region, shown dependence on waist diameter; (**b**) C-Band, displacement of multiple plasmons with refractive index (figures originally published in [10] and [9], ^©^ 2010 and 2014, reproduced with permission from Elsevier).

In Figure 5 we show results for visible and C-Band, and in Table 1 we summarize the characteristics parameters of DLUWTs when used in the different spectral regions. Note how high the sensitivities of these devices are.

## 6. Experimental Results III: Variations and Improvements

Many other different possibilities have been tested with DLUWTs as SPR sensors starting from the basic scheme depicted above. We summarize some of them here.

### 6.1. Use of InN as Dielectric Material

**Figure 6.**
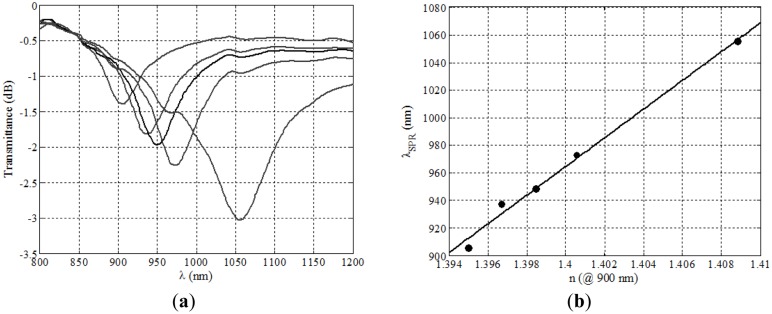
(**a**) Behaviour of a DLUWT with 8 nm of aluminum and 30 nm of indium nitride. Sensitivity, measured from the slope of the curve of figure (**b**) is 10,800 nm/RIU (figures originally published in [12], ^©^2011, reproduced with permission from Elsevier).

The use of InN implies some advantages from the fabrication point of view and it is also an environmental-friendly material. In principle, we can consider it as one of many candidates to assume the role that TiO_2_ has been playing, but, preliminary as they are, the experimental results obtained show how incredibly high the sensitivity of InN-based devices is (without any other change in the usual components of DLUWTs). In Figure 6 we show the behavior of an asymmetric DLUWT with an InN layer [12].

### 6.2. Pre-Treatment of Fibers with Chemical Attack

The chemical attack to the fibers with HF induces some roughness on the surfaces. In principle, it is expected that the deposition is favored, and, effectively, it contributes to the stability of the behavior of the devices in repeated immersions. Also, the variability of thickness associated to the roughness diminishes the dependence of the devices with polarization and contribute to the disappearance of secondary plasmon dips, thus approaching the behavior of asymmetric devices to that of symmetric ones [13].

### 6.3. Reflective Measuring Configuration

Polishing the end of the fiber after the tapered region and using a silver deposition technique we can transform our devices, that usually operate in transmissive configuration, into reflective-mode transducers, making them more compact and smaller and simpler to operate in real measurement conditions. The double pass through the interaction region increases the response and decreases polarization dependence [14].

### 6.4. Interrogation Scheme Based on FBGs

**Figure 7.**
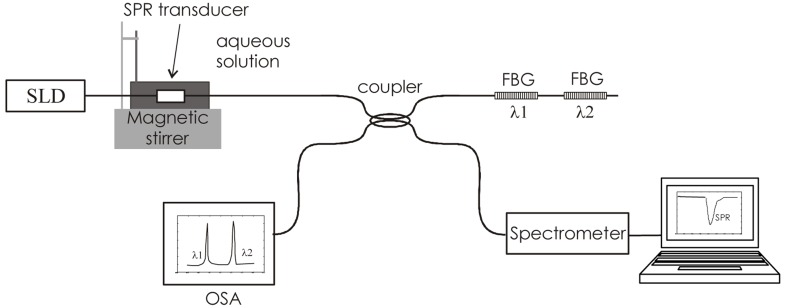
Scheme of an advanced interrogation technique for SPR sensors based on DLUWTs and FBGs (figure originally published in [15], ^©^ 2010, reproduced with permission from Elsevier).

With the intention of pushing the limits of SPR devices as far as we can, and also to provide the possibility of self-referencing and/or independence with temperature, we propose a new concept of sensor that, while still based on DLUWTs, incorporates fiber-Bragg gratings (FBGs) not as part of the transduction, but of the interrogation (Figure 7). This is a powerful concept, when considering the extreme spectral sensitivity of FBGs and the fact that most of the SPR-based measurements are related to the determination of the displacement of a given wavelength, associated to a resonance. In Figure 7 we show a scheme of the proposed setup. The combination of the signals of the gratings permits to increase the resolution of the measurements. These improvements are very easily implemented when working with such simple devices as DLUWTs [15].

### 6.5. Selectivity through Absorption

All SPR refractometers (those presented here and also any other one, before any recognition agent is added) measure only variations of the refractive index of the outer medium, independently of how these variations are produced. This means that they are not selective. They begin to be selective when we choose a substance to be *added* to the basic SPR sensor setup that reacts to the analyte in any or other way. But we have showed, first theoretically [16] and then experimentally [17] that a conventional SPR device (one of the DLUWTs presented here with no further modification whatsoever) is in fact selective if the outer medium is absorptive and we tune the position of one of the plasmon wavelengths to one of the absorption wavelengths of that medium. The effect of this coupling is a, so to speak, inhibition of the plasmon, with an increase on the value of the transmittance corresponding to that dip, while the usual variation of the real part of the refractive index still produces the displacement of that dip.

**Figure 8.**
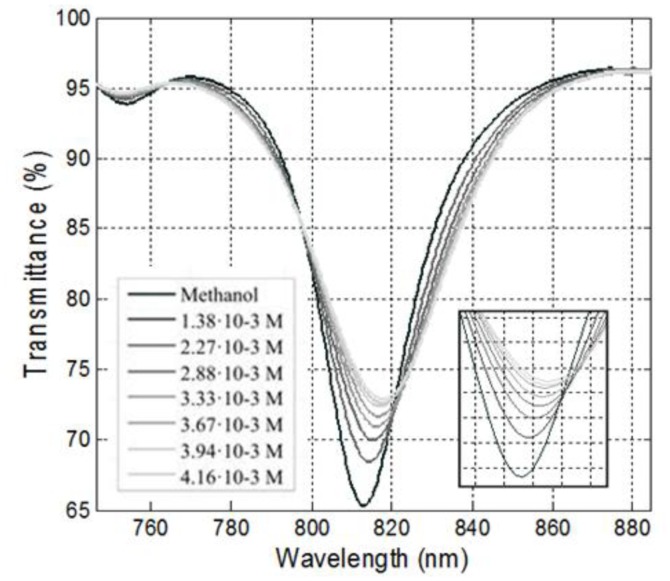
Behavior of a DLUWT when the resonance is tuned to an absorption wavelength of a substance present in the outer medium in terms of its concentration (figure originally published in [17], ^©^ 2011, reproduced with permission from Elsevier).

This is a very interesting effect, both from the theoretical and experimental point of view, and, although it can be applied with any scheme of plasmon excitation, it is with DLUWTs that it finds its real value, due to the versatility of these devices, and the possibility of tuning of their resonances in an easy way, as depicted above. This is an ongoing research and will become more fruitful in the future. We show the effect of the “plasmon inhibition” for absorptive medium in Figure 8. This effect is, of course, increased with the concentration of the absorptive species that can, in this way, be measured.

## 7. Conclusions/Outlook

We have presented in this review a brief summary of the main results concerning the use of doubly deposited uniform-waist tapered optical fibers (DLUWTs) as surface plasmon resonance (SPR) transducers. DLUWTs are easily fabricated and their construction parameters can be accurately controlled. The thicknesses of the layers can be varied to adapt the response of the sensors to different wavelength ranges, from the visible to the C-band in the infrared. In all cases, the systems show high sensitivity and very good general performance.

DLUWTs also provide unique features, that cannot be found in other SPR devices. For instance, asymmetric deposits produce multiple plasmon dips that can be used for simultaneous measurement of different effects or for self-referencing. Also, the dependence on polarization of DLUWT-based SPR transducers is small, and can even be eliminated with symmetrical deposits.

DLUWTs can be used as a part of ambitious, sophisticated interrogation schemes involving FBGs, and their performance can be improved also by chemical preprocessing of the surfaces of the fibers. Additionally, when used in absorptive media they can provide selectivity to specific chemical species without the need of recognizing agents.

For all these reason, tapered-fiber-based SPR transducers are ideal for their use as basis for chemo- and biosensors for exigent measurement conditions. The main challenge now is to adapt the basic scheme here presented to specific analyte detection, to ensure the correct incorporation of chemical reactants to the taper.

Also, the good performance of tapered-fiber-based SPR refractometers permits to think in the determination of different physical parameters via the coupling with refractive index variation, for instance, through magneto-optical or electro-optical effects. Finally, the dynamics of plasma-wave excitation and propagation in guiding structures is also greatly clarified with the experimental results here exposed.

## Figures and Tables

**Table 1.** Summary of SPR sensors based on DLUWTs for different spectral regions (table originally published in [10], ^©^ 2014, reproduced with permission from Elsevier).

**Wavelength Range**	**Thickness of Al [nm]**	**Thickness of TiO_2_[nm]**	**Sensitivity [nm/RIU]**	**Ref.**
550–700 nm	8	25	∼2000	[10]
750–850 nm	8	60	∼4000	[8]
C-Band, ∼1.5 μm	19	99	∼5000	[9]
